# Electronic Mentoring Programs and Interventions for Children and Youth With Disabilities: Systematic Review

**DOI:** 10.2196/11679

**Published:** 2018-10-24

**Authors:** Sally Lindsay, Kendall Kolne, Elaine Cagliostro

**Affiliations:** 1 Bloorview Research Institute Holland Bloorview Kids Rehabilitation Hospital Toronto, ON Canada; 2 Department of Occupational Science and Occupational Therapy University of Toronto Toronto, ON Canada

**Keywords:** youth, disability, eHealth, mentoring, review, peer support, adolescent, child, disabled children, disabled persons, telemedicine

## Abstract

**Background:**

Children and youth with disabilities experience many challenges in their development, including higher risk of poor self-esteem, fewer friendships, and social isolation. Electronic mentoring is a potentially viable approach for youth with disabilities to access social and peer support within a format that reduces physical barriers to accessing mentors.

**Objective:**

Our objective was to synthesize and review the literature on the impact of electronic mentoring for children and youth with disabilities.

**Methods:**

We conducted a systematic review, completing comprehensive searches of 7 databases from 1993 to May 2018. We selected articles for inclusion that were peer-reviewed publications, had a sample of children or youth with disabilities (≤25 years of age), and had empirical findings with at least one outcome focusing on the impact of electronic mentoring. Two reviewers independently applied the inclusion criteria, extracted the data, and rated the study quality before discussing the findings.

**Results:**

In the 25 studies meeting our inclusion criteria, 897 participants (aged 12-26, mean 17.4 years) were represented across 6 countries. Although the outcomes varied across the studies, of 11 studies testing significance, 9 (81%) reported a significant improvement in at least one of the following: career decision making, self-determination, self-advocacy, self-confidence, self-management, social skills, attitude toward disability, and coping with daily life. The electronic mentoring interventions varied in their delivery format and involved 1 or more of the following: interactive websites, virtual environment, email, mobile apps, Skype video calls, and phone calls. A total of 13 studies involved one-to-one mentoring, 6 had group-based mentoring, and 6 had a combination of both.

**Conclusions:**

The evidence in this review suggests it is possible that electronic mentoring is effective for children and youth with disabilities. More rigorously designed studies are needed to understand the impact and effective components of electronic mentoring interventions.

## Introduction

### Background

Approximately 3.7% of Canadian children [1] and 5.6% of American children [2] have a disability and encounter many challenges to their full participation and inclusion in society. They are frequently socially isolated, physically excluded, and at risk of abuse and poor developmental, social, and vocational outcomes [3,4]. Further, they are often less well equipped with emotional, social, and cognitive resources to achieve positive life outcomes [3,5]. Youth with disabilities are underrepresented in higher education and have a lower likelihood of completing school than do youth without disabilities [6,7]. These trends are often a result of negative attitudes, discrimination, inaccessible environments, and lack of resources and social supports [8-10]. Focusing on children and youth with disabilities is critical because disadvantages are compounded for those who start life with a disability [11,12]. They are a unique population that often encounters multiple disadvantages, particularly with developmental tasks, social development, and role functioning [13,14].

Mentoring is a promising mechanism that could help to enhance youth’s inclusion in society [15-18], while offering support and coping strategies [19]. Mentoring involves a relationship between a more experienced individual, who serves as a role model and shares his or her experiences, and a less experienced individual [17,20,21]. Mentoring can offer informational, practical, and emotional assistance along with coping skills [16,22-24]. Until recently, most mentoring programs have not included or specifically targeted youth with disabilities [16,25]. Having mentors for youth with disabilities is critical for developing their social capital, self-determination, quality of life, and career development goals [16,17,26-29]. Research focusing on face-to-face mentoring for youth with disabilities shows beneficial impacts on transition to postsecondary education and employment [16,26], social competence and self-esteem [30], and independent living skills [11].

While there are benefits to face-to-face mentoring for improving transitions to school or employment for youth with disabilities, there are challenges associated with this type of mentorship, including difficulty in finding and accessing mentors [16]. Electronic mentoring (e-mentoring), defined as a “computer mediated, mutually beneficial relationship between a mentor and a protégé which provides learning, advising, encouraging, promoting and modeling that is often boundaryless, egalitarian and qualitatively different than traditional face-to-face mentoring” [31], can help to overcome some of these challenges. Computer-mediated communication has helped to advance e-mentoring as a promising mode of developing mentoring relationships and changing the conditions under which mentoring is sought and offered [31]. Another potential advantage of e-mentoring is that it offers a viable platform for increasing the availability and accessibility of mentors [32].

Consistent evidence shows that Web-based platforms and mobile apps can influence learning and behavior change [12,33-39]. Given that technology is already an important component of adolescents’ social networks, whereby most youth seek information and communicate over the internet and approximately 88% of American teenagers have a mobile phone [40], e-mentoring interventions are a promising approach to helping youth with disabilities. This mode of mentoring has the potential to enhance social support while reducing barriers because of differences in sex, ethnicity, disability, or geographic location [14,41,42]. People with mobility issues or speech, hearing, or vision difficulties can participate when using appropriate adaptive devices [43].

There are barriers associated with traditional, face-to-face mentoring, limiting the full participation of certain groups, including youth with disabilities. The Web-based, electronic delivery format offered by e-mentoring can help to make mentoring relationships more accessible and available to groups that have had limited access to mentoring [31]. Electronic communication allows for flexibility in matching partners and asynchronous communication [44]. Research shows that e-mentoring has many of the same benefits as face-to-face mentoring, including informational, psychosocial, and instrumental benefits [45]. Among youth without disabilities, Web-based support has been shown to predict a lower incidence of depressive thoughts and can buffer the effects of peer victimization [46].

Challenges associated with the implementation of e-mentoring programs include access to technology, computer literacy, and adequate communication skills [16,31,47]. Further, finding the right mentors, developing a rapport, and keeping participants engaged can be difficult, especially if they have never met face-to-face [44,48]. Miscommunications can also occur because Web-based interactions conceal social cues and inhibit communication [31,49].

### Objectives

E-mentoring provides an opportunity to “level the playing-field...for those who otherwise would be left out of important informal networks” [45], including youth with disabilities. Although there has been an increase in e-mentoring research for youth with disabilities, this work has not yet been synthesized. This systematic review of e-mentoring research is an important step in identifying the common components of e-mentoring interventions and developing an understanding of the effectiveness of this approach for youth with disabilities. Our specific objectives were to (1) critically appraise and synthesize the peer-reviewed evidence on e-mentoring for children and youth with disabilities, and (2) highlight gaps in understanding and areas for future research.

## Methods

### Rationale for a Systematic Review

We conducted a systematic review because a meta-analysis was not feasible given the heterogeneity of the studies reviewed (eg, various disability types, study populations, interventions, and outcome measures). Systematic reviews are the next highest level of evidence aiming to critically appraise the evidence of e-mentoring for children with disabilities and provide an unbiased summary of current practices [50].

### Search Strategy and Data Sources

**Figure 1:**
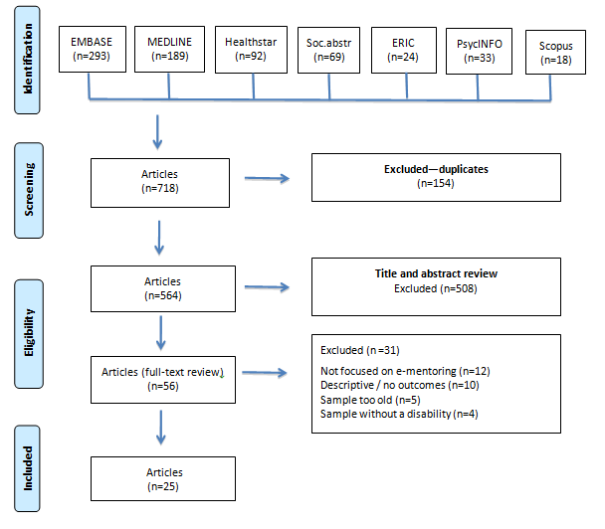
Search process flow diagram. ERIC: Education Resources Information Center; Soc.abstr: Sociological Abstracts.

We conducted a comprehensive search of the published peer-reviewed literature using the following databases: MEDLINE, EMBASE, Healthstar, Sociological Abstracts, Education Resources Information Center (ERIC), PsycINFO, and Scopus (see Figure 1). We searched for headings and key terms related to mentoring, electronic and computer-mediated forms of mentoring, disability, and youth (see Multimedia Appendix 1 for full list of terms). We searched for articles published between 1993 and May 2018. We also manually searched the reference lists of all articles meeting our inclusion criteria.

### Article Selection

To select articles for the review, we applied the following inclusion and exclusion criteria. Eligibility criteria were (1) publication in a peer-reviewed journal between 1993 and May 2018, (2) study population focusing on children or youth (aged ≤25 years) with a disability (eg, physical, developmental, intellectual, or sensory), and (3) focus on e-mentoring (defined as computer-mediated technology, such as the internet, mobile apps, or Skype). We excluded articles that (1) were not peer reviewed (eg, opinion, editorial, gray literature, or reports) and (2) focused on descriptions of e-mentoring programs that did not have empirical findings.

Our initial search identified 718 articles for potential inclusion in this review (see Figure 1). After removing the duplicates, 2 authors independently reviewed the titles and abstracts for inclusion. A total of 508 abstracts did not meet our inclusion criteria. We read the remaining 56 articles and independently applied the inclusion criteria. Of these, 25 articles met our inclusion criteria. We kept field notes of our inclusion and exclusion decisions and discussed among the team any discrepancies on which articles were to be included.

### Data Abstraction and Synthesis

The first author (SL) extracted and compiled the data from the 25 articles selected for review using a structured abstraction form. She abstracted relevant information on each study (eg, year, country, objectives, disability type, study design, intervention, key findings, quality appraisal score, and limitations; see Multimedia Appendix 2). All 3 authors reviewed all 25 articles and abstracted data for accuracy. We noted the limitations and risk of bias for each study.

We synthesized our findings based on the guidelines for narrative synthesis [51], which is relevant for reviews with diverse methodologies. Our method of synthesis involved a structured examination and summary of all studies included in the review. Our first step organized the studies into categories to help guide the analysis. Next, we explored within-study findings through a narrative description of each study’s results while considering the quality and rigor of the design. Our next step involved undertaking a cross-study synthesis while considering variations in study participants and design [51].

### Methodological Quality Assessment

Our findings and recommendations for future research are based on the overall strength and quality of the evidence we reviewed. The measure of bias and quality assessments were based on the American Academy of Neurology’ guidelines [52] to assess interventions and randomized controlled trials (RCTs), and Kmet and colleagues’ [53] standard quality assessment and risk of bias (for all other quantitative and qualitative studies). The American Academy of Neurology guidelines are a widely recognized tool for therapeutic interventions to help inform appropriate recommendations for interventions [52]. All 3 authors independently reviewed each article and assigned a score for each item and an overall score.

All 3 authors independently applied a 14-item checklist for quantitative studies and a 10-item checklist for qualitative studies [53] to help assess the quality of evidence for each study. Multimedia Appendix 3 and Multimedia Appendix 4 show the results of the quality assessments. We did not exclude any studies from our review based on quality. We followed the Preferred Reporting Items for Systematic Reviews and Meta-Analyses, a method of transparent reporting [54]. We noted any issues concerning study limitations or risk of bias. Any discrepancies in the ratings were resolved through discussion and reexamining the article.

## Results

### Study and Participant Characteristics

A total of 25 articles met our inclusion criteria for this systematic review (see Figure 1). Of these, 14 studies were conducted in the United States, 6 in Canada, 2 in the Netherlands, 1 in Australia, 1 in Israel, and 1 in South Korea. Reported sample sizes of the mentees ranged from 1 to 189, for a total of 897 mentee participants. Ages of the participants ranged from 12 to 26 years (mean 17.4 years). The types of disabilities these studies focused on were rheumatic disease, juvenile arthritis, cerebral palsy, spina bifida, muscular dystrophy, pediatric transplant, blindness or vision impairments, chronic pain, traumatic brain injury, and various other types (eg, vision, hearing, learning, or developmental disabilities).

### Outcome Measures

Several standardized and nonstandardized measures were used to explore the role of e-mentoring for youth with disabilities. Studies that used standardized measures assessed the Dutch arthritis self-efficacy scale, self-management, self-determination, self-advocacy, career decision self-efficacy, mathematics self-advocacy, self-confidence, quality of life, the Miller Hope Scale, sense of community, self-perception profile, pain inventory, daily functioning, and children’s inventory of social support. Nonstandardized measures assessed perceived usefulness, use, user acceptance, satisfaction, fidelity, engagement, feasibility, acceptability, medication use, adherence, social support, social behavior and functioning, loneliness, self-reported coping, pain, frequency of communication, employment, and program likes and dislikes.

### Methodological Design and Theoretical Frameworks

The methodological designs varied across the studies and included 3 RCTs, 7 surveys, 1 case study, and 1 feasibility study. Of the 25 studies, 12 (48%) had a theoretical framework such as self-efficacy theory, social support theory, electronic socioemotional support theory, conceptual framework for peer mentoring support in health care, self-determination theory, social learning theory, symbolic interaction theory, and theory of change.

### Intervention Components

#### Mode of Delivery

The e-mentoring interventions varied in their delivery format and involved 1 or more of the following: interactive websites [13,55-60], virtual environment [27,61-63], email [19,47,64-71], mobile apps [60,63,72], Skype video calls [60,73-75], and phone calls [68,71]. Overall, studies involving e-mentoring apps reported they were a feasible and helpful tool for facilitating students’ understanding [60,72]. Studies including email-based mentorship programs reported improved mentor-mentee communication [64,65,70], particularly with respect to personal, more informal communication [19,47,70]. When compared with face-to-face mentoring, e-mentoring through interactive websites had similar outcomes for self-efficacy, quality of life, and self-management [55] and for dealing with daily life [57], but were reported to be feasible [57,60] and fun [13] while providing a safe environment for socialization [58]. Skype-based mentorship programs were feasible [73], provided flexibility [74], were informational, and provided appraisal and emotional support [75]. Studies described that e-mentoring through virtual environments facilitated improvements in self-determination and self-advocacy [27,62], engagement [61], and persistence [27,62,63].

#### Mentoring Format

A total of 13 studies involved one-to-one mentoring [27,47,57,60,65-68,71-76], 6 had group-based mentoring [13,55,57-59,61], and 6 had a combination of both one-to-one and group mentoring [56,62-64,69,70]. No clear pattern emerged regarding differential benefits for one-to-one versus group-based mentoring programs.

#### Types of Mentors

The types of mentors and the training they received varied across the studies. Interestingly, the definition of mentor was used broadly across studies, particularly in what was considered a “more experienced individual.” In total, 12 studies involved a mentor who had a similar type of disability to the mentee [13,47,55,57,59,65,66,68,71,73,74,77], whereas 2 studies had mentors who were near-peers without disabilities [60,61]. Meanwhile, 7 had adult mentors without disabilities [27,62-64,67,69,70], including 3 studies that defined clinicians [56,58,76] and a writing coach [72] as mentors. Positive effects of e-mentoring were reported for all types of mentors but, given the heterogeneity of outcomes, it was not possible to compare the effectiveness of types of mentors across studies.

#### Intervention Dosage

Of the 25 studies, 10 (40%) provided sufficient information to calculate the intervention dosage. The overall duration and dosage of the interventions in our review ranged from 0.31 hours per week to 2 hours per week, occurring over a period of 4 to 24 weeks. Of the 25 studies, 11 (44%) did not provide information on the hourly dosage of the intervention; however, information on the length of exposure was provided. For these studies, exposure ranged from 2 months to 4 years. No clear pattern emerged regarding the length of the intervention, and both shorter and longer interventions reported positive results. For example, Stinson et al [74] reported a relatively short intervention (2.5-5 hours) that was engaging and led to increased self-management ability, while Stewart et al [59] conducted a longer intervention (25-37.5 hours) and reported decreased loneliness, increased acceptance, and greater self-confidence.

### Effectiveness of the Interventions

Although the outcomes of the interventions varied across the studies, of 11 studies testing significance, 9 (81%) reported that mentoring helped improve outcomes. For example, the studies in our review found that mentoring significantly improved career decision making (large effect) [68], personal hope for the future [68], positive attitudes about disability (large effect) [68], coping with daily life [57], self-determination [62], self-advocacy [63,69], self-confidence [59], career decision self-efficacy [77], self-management skills [73,74], mathematics self-efficacy [62], social skills [69], social contact [59], loneliness (decrease) [59], social and behavioral functioning [60], and written communication [67]. It is important to note that 1 study found that mentoring made no significant difference in self-efficacy, quality of life, and self-management [55]. Other improvements (not reporting significance tests) included the following: transition toward using augmentative and alternative communication devices [65]; science, technology, and mathematics learning and emotional supports [27]; persistence in science, technology, and mathematics education [63]; understanding of changes needed for school work [72]; informational, appraisal, and emotional support [75]; and understanding of colleges, majors, and admissions [77].

### Outcomes by Level of Evidence

Multimedia Appendix 5 provides an overview of the e-mentoring intervention outcomes by level of evidence, classifying the studies according to the American Academy of Neurology guidelines [52]. We classified 3 studies were classified as level 1 (ie, rigorous RCT), 1 of which involved an interactive, group-based self-management website [55] for youth aged 17 to 25 years with rheumatic disease. Mentors were peer leaders who had the same condition. This RCT found no significant differences in self-efficacy, quality of life, or self-management between experimental and control groups [55]. Meanwhile, 2 level 1 studies involved one-to-one Skype calls [73,74]. Ahola Kohut and colleagues’ study involved youth aged 12 to 18 years with chronic pain and mentors with a similar condition [73]. They engaged in 10 one-to-one Skype video calls (using iPeer2Peer). Their RCT found that the intervention was feasible and acceptable, with significant improvements in self-management skills and coping [73]. Stinson et al [74] used 10 one-to-one Skype-based video calls (iPeer2Peer) for youth aged 12 to 18 years with juvenile arthritis involving mentors with a similar condition. Their RCT found significant improvements in perceived ability to manage arthritis compared with controls. Participants were satisfied with the intervention and stated that they would recommend it to peers.

Only 1 study in this review was a level 2 (ie, matched cohort study, or RCT in a representative sample lacking 1 criterion in level 1) [52]. This mixed-methods prospective cohort study involved an online mentor with group-based and one-to-one components for youth with various types of physical and developmental disabilities [56]. Their findings showed that the utility of the intervention was modest, and only 20 of 50 (40%) participants engaged in chats with the mentor [56]. It was interesting to note that their mentor was a clinician (ie, occupational therapist) and not a youth.

None of the studies was a level 3 (ie, all other controlled trials). Meanwhile, 21 studies in our review were a level 4 (ie, all other studies), which had a wide range of outcomes (see Multimedia Appendix 5). Of the level 4 studies, 8 used an email mentoring approach [47,64-69,78] among youth with blindness, learning and cognitive disabilities, and various other types of disabilities (vision, hearing, and learning). These studies reported improvements in the transition to using assistive devices [65], career decision self-efficacy [68], attitudes about disability [68], preparation for college and employment [69], and written communication [67].

A total of 5 level 4 studies [13,57-59,66] used an interactive, group-based website in their approach to mentoring youth with cerebral palsy, spina bifida, juvenile arthritis, muscular dystrophy, and other physical, behavioral, and intellectual disabilities. Of these, 3 reported that their intervention was feasible [13,57,66], helped participants to cope with daily life [57], provided a space for socialization [58], and decreased loneliness while increasing acceptance, confidence, and a sense of community [59].

Of the level 4 studies, 4 used a virtual-world approach [27,61-63] and involved youth with various types of disabilities (ie, learning, visual, and physical disabilities, autism, and transplant recipients). These studies reported that their intervention helped to improve engagement and psychoeducational goals [27]; enhanced science, technology, and mathematics learning and emotional supports [62]; and improved persistence in science, technology, and mathematics education and self-advocacy [27,63].

A mobile app was used in 2 level 4 studies for their mentoring approach for youth with cerebral palsy [72] and traumatic brain injury [60]. These interventions were feasible and acceptable to participants [60,72], helped youth to define and achieve goals [60], improved social and behavioral functioning [60], and improved understanding of school work [72].

One level 4 study used Skype video calls of 1 hour a week for 4 weeks for their mentoring intervention and involved youth with various disability types [60]. This intervention was reported to be feasible and acceptable to participants [60,73,74], and helped youth achieve goals.

Based on the evidence in this review and using the American Academy of Neurology guidelines [52], we found that e-mentoring interventions are possibly effective or useful for this population. This rating is based on the overall rigor of the studies and the strength of the evidence [52]. Among the reviewed studies, 8 reported on the feasibility and acceptability of the program, whereby participants found that the mentoring format was favorable [13,57,60,64,66,71,73,74]; 1 study found that the utility of their Web-based intervention was modest [56].

### Moderating Factors

#### Sex of Participants

A total of 4 studies noted sex differences in their mentoring program. Specifically, Barnfather et al [13] found that female participants with cerebral palsy or spina bifida were significantly more likely than male participants to contribute to the online discussion. Burgstahler and Doyle [70] reported that male participants sought and provided information about technology and the internet, while female participants communicated more frequently and shared more personal information. Another study found the greatest improvements in a science, technology, and mathematics e-mentoring program among female participants [27]. In Parkyn and Coveney’s [58] e-mentoring program among youth with muscular dystrophy, they found that the intervention had a strong collective identity reflecting ideals of masculinity.

#### Communication and Relationship Development

One study exploring peer-to-peer and mentor-protégé relationships found that, although they performed similar functions, peer-to-peer relationships were more personal [64]. Burgstahler and Cronhiem [64] reported that barriers to an e-mentoring format included difficulty expressing feelings, dealing with lots of messages, and technical difficulties (eg, losing an internet connection or the website not working properly).

Another moderating factor reported by Cantrell et al [61] is the ability of the mentors themselves to develop relationships with participants and keep them engaged. Cohen and Light [65] similarly discovered that the frequency and length of communications between mentors and mentees may have been influenced by the availability of mentors and the quality of the match. Another study found that successful mentoring included an informal and supportive style, whereas unsuccessful mentoring was linked with a formal style [47]. Other moderating factors influencing the utility of the mentoring program included typing speed, cognitive skills, and need for support [13].

The results from qualitative studies highlight the perceptions and feelings of participants toward e-mentoring and the aspects they found successful, and the themes of discussion within mentoring sessions. E-mentoring was found to be a favorable environment [64] and a safe opportunity for socialization [58]. Barriers to successful mentoring included difficulty expressing feelings, lots of messages, technical difficulties, and a more formal communication style with a distant tone [47,64]. Themes in online mentored discussions included illness impact, self-management, non-illness–related goals, hobbies and social environments, bullying, physical appearance, school, and pain management [61,75]. Overall, the qualitative findings suggest that e-mentoring is an effective method for reducing the barriers associated with face-to-face mentoring [19,62,64,65,72] and facilitating the mentor-mentee relationship, particularly through informal communication [47,64].

### Quality Assessment and Risk of Bias Within Studies

Two authors (SL, KK) independently rated the quality of each study using American Academy of Neurology guidelines (for levels 1-2) and Kmet and colleagues’ [53] standard quality assessment (for all level 4 studies). Total scores for the quantitative studies range from 33% to 96% (mean 69.8%; Multimedia Appendix 3) and 35% to 85% for the qualitative studies (mean 72.2%; Multimedia Appendix 4). It is important to note that the quality assessments measure different items based on the qualitative or quantitative nature of the study. Most discrepancies reflected the extent of the applicability of each of the items. These articles were read and scored again by a third author (EC) and discussed until consensus was reached.

We carefully examined the limitations, quality, and risk of bias within each study. Areas of the quality assessment where quantitative studies scored lower included controlling for confounding and reporting estimates of variance. For most of the quantitative studies, random allocation and blinding of investigators and participants was not possible. For the qualitative studies, areas of the quality assessment scoring lower included the description of the sampling strategy and reflexivity of the account.

### Risk of Bias Across Studies

We considered the risk of bias across the studies in our review. First, the studies were done in 6 countries, all which have varying programs and policies related to mentoring. Second, we included various types of disabilities and a wide age span and, therefore, caution should be used in generalizing the findings. Third, many of the studies had different interventions (eg, email, Skype, interactive website) and components (eg, length of program, type of mentor, one-to-one or group based), and it is unclear which components contributed to what outcomes. Fourth, although we consulted a librarian to help design our search strategy, it is possible we may have missed some articles. Fifth, various unstandardized measures were used, which limited our ability to compare across interventions.

## Discussion

### Principal Findings

This review explored e-mentoring interventions for children and youth with disabilities over a 25-year period. Although the outcomes varied across the studies, of 11 studies testing significance, 9 (81%) reported a significant improvement in at least one of the following: career decision making, self- determination, self-advocacy, self-confidence, self-management, social skills, attitude toward disability, and coping with daily life. Exploring this topic is important because youth with disabilities are at a higher risk of social isolation, discrimination, peer victimization, and poorer academic, social, and vocational outcomes than are youth without disabilities [4,8,9]. E-mentoring is a potentially viable approach offering a form of social support while overcoming challenges in accessing mentors in a face-to-face format [14].

Based on our assessment of the overall rigor of these studies and the strength of the evidence, we find that e-mentoring interventions are a possibly effective tool for youth with disabilities. These results are important because research shows that youth with disabilities encounter perpetual barriers and discrimination in participating in everyday activities [8,9]. Helping youth to develop their self-determination and self-advocacy skills is important because it is critical for optimizing an individual’s participation and inclusion in society [79-81].

### Program Design Implications

Our review highlights that several different types of e-mentoring interventions have the potential to positively influence youth with disabilities. Common components of programs showing improved outcomes included interactive website, Skype calls, virtual world, email, and mobile apps. These findings corroborate studies of programs for youth without disabilities reporting advantages to e-mentoring programs, including a range of delivery formats, such as email, face-to-face meetings, telephone calls, and video conferencing [45]. The benefits of e-mentoring programs for youth without disabilities are similar to those reported here and include informational, psychosocial (eg, improved self-esteem, increased confidence, and greater support for risk taking), and instrumental benefits for creating opportunities for the mentee [45]. These benefits are mirrored in our findings for youth with disabilities, where studies reported increased self-determination, self-advocacy, and self-confidence associated with e-mentoring programs. In our review, additional social benefits of e-mentorship included decreased loneliness, improved social skills, and increased social contact, supporting career decision making and improving positive attitudes toward their disability. The additional advantages of e-mentoring for youth with disabilities further the utility of this format for reducing the barriers associated with face-to-face mentoring and increasing the availability and accessibility of mentoring relationships.

### Research Implications

Although the majority of the studies in our review had diverse samples, most did not explore any differences in outcomes based on sociodemographic characteristics of the participants, especially disability type. Many studies included participants with multiple disability types, and in these cases, differences in the effect of the intervention between disability types were not examined. It is possible that e-mentoring interventions provide differential benefits based on the specific needs of a given disability type; however, this was often not explored. Additionally, there was surprisingly little discussion within the studies on access to technology and whether youth needed to use assistive devices or other supports to participate. Addressing the impact of access to technology is crucial in the context of the digital divide, where inequities in access [82] are often explained by socioeconomic gaps in technology [82]. Research suggests that information technology can help provide people with disabilities with a set of tools to enable their participation and personal development [83,84]. It is important to consider that the cost of adapted technology and assistive technology can be expensive and a potential barrier for people with disabilities accessing the internet, mobile apps, etc [83].

It was interesting that 4 studies explored the role of sex and reported differences in the communication style of youth and their engagement in mentoring programs. These findings have implications for the broader field of research on mentoring, specifically on the relationship between a person’s sex and mentoring. Previous work shows that females perceive more barriers to mentorship than males and have more difficulty finding mentoring relationships [85]. However, there is a lack of research comparing the ways in which males and females engage in mentoring relationships and how these interactions influence the effectiveness of mentorship programs.

### Future Directions and Implications for Practice

Although our review revealed promising benefits from e-mentoring interventions, more research is needed to explore what types of interventions work best for whom and the optimal delivery formats. Further research is needed to explore whether different delivery formats affect outcomes. Future studies should consider what type of mentor (eg, near-peer with a disability, adult mentor, or youth without a disability) is linked with the optimal outcomes. More rigorous designs are needed to explore formats that were explored only in level 4 studies (eg, email mentoring, group-based interactive websites, virtual world approaches, mobile apps, and Skype video calls). Future research should examine the relationship between socioeconomic and other demographic factors and access to technology in the development of e-mentoring programs for youth with disabilities. Further studies should consider investigating the impact of a person’s sex on the ways in which youth with disabilities use e-mentoring programs, comparing potential sex-based difference in effectiveness of interventions, communication, and engagement. Such research is important for increasing the engagement of women in science, technology, and mathematics fields, where both women and individuals with disabilities have long been underrepresented [70]. Finally, more research needs to explore the influence of socioeconomic status on the use of e-mentoring programs and the impact of the digital divide on the accessibility of information communication technology for youth with disabilities participating in e-mentoring programs.

Those designing and implementing e-mentoring programs for youth with disabilities should consider moderating factors such as type of disability, sex, and communication style. Given that many studies demonstrated that various forms of e-mentoring are feasible and acceptable to participants, the next steps should be to consider how to scale up these interventions to larger numbers of participants and various types of disabilities.

### Conclusions

The results of this review suggest that e-mentoring is a potentially viable method for improving the accessibility and availability of mentors for children and youth with disabilities. The interventions we reviewed were found to be feasible and useful, and facilitated improved communication in mentor-mentee relationships. The studies reported a range of benefits that addressed many of the challenges faced by individuals with disabilities throughout development, including reduced social isolation, increased self-confidence and self-efficacy, and improved career readiness and decision making. Further studies with more rigorous design are required to identify and compare the effectiveness of the components of e-mentoring interventions (eg, type of mentor, group or one-to-one mentoring, and format of delivery), as well as to understand the influence of the mentee’s sociodemographic factors (eg, type of disability, sex, and socioeconomic status) on the impact of e-mentoring programs.

## Appendix

Multimedia Appendix 1 Search strategy.

Multimedia Appendix 2 Overview of studies.

Multimedia Appendix 3 Scores using the standard assessment criteria for quantitative studies.

Multimedia Appendix 4 Scores using the standard assessment criteria for qualitative studies.

Multimedia Appendix 5 Overview of components of e-mentoring interventions by level of evidence.

## Abbreviations

e-mentoring: electronic mentoring
ERIC: Education Resources Information Center
RCT: randomized controlled trial

## References

[1] Statistics Canada (2006). Disability in Canada: A 2006 Profile.

[2] Yang L, Hock T (2016). Disability statistics.

[3] McDonald KE, Balcazar FE, Keys CB, DuBois DL, Karcher MJ. (2005). Youth with disabilities. Handbook of Youth Mentoring.

[4] Lindsay S, McDougall C, Menna-Dack D, Sanford R, Adams T (2015). An ecological approach to understanding barriers to employment for youth with disabilities compared to their typically developing peers: views of youth, employers, and job counselors. Disabil Rehabil.

[5] United Nations Children's Fund (2013). State of the world's children 2013: children with disabilities.

[6] Organisation for Economic Co-operation and Development (2015). Education at a Glance 2015: OECD Indicators.

[7] World Health Organization (2015). Disabilities.

[8] Lindsay S, McPherson AC (2012). Experiences of social exclusion and bullying at school among children and youth with cerebral palsy. Disabil Rehabil.

[9] Lindsay S (2011). Discrimination and other barriers to employment for teens and young adults with disabilities. Disabil Rehabil.

[10] Lindsay S, Yantzi N (2014). Weather, disability, vulnerability, and resilience: exploring how youth with physical disabilities experience winter. Disabil Rehabil.

[11] West M, Targett P, Steininger G, Anglin N (2001). Project Corporate Support (CORPS): a model demonstration project on workplace supports. J Vocat Rehabil.

[12] Lindsay S, Smith S, Bell F, Bellaby P (2007). Tackling the digital divide: exploring the impact of ICT on managing heart conditions in a deprived area. Inf Commun Soc.

[13] Barnfather A, Stewart M, Magill-Evans J, Ray L, Letourneau N (2011). Computer-mediated support for adolescents with cerebral palsy or spina bifida. Comput Informatics Nurs.

[14] Lindsay S, Stinson J, Stergiou-Kita M, Leck J (2017). Improving transition to employment for youth with physical disabilities: protocol for a peer electronic mentoring intervention. JMIR Res Protoc.

[15] Burgstahler S, Crawford L (2007). Managing an e-mentoring community to support students with disabilities: a case study. Assoc Adv Comput Educ J.

[16] Lindsay S, Hartman L, Fellin M (2016). A systematic review of mentorship programs to facilitate transition to post-secondary education and employment for youth and young adults with disabilities. Disabil Rehabil.

[17] Stumbo N, Martin J, Nordstrom D, Rolfe T, Burgstahler S, Whitney J, Langley-Turnbaugh S, Lovewell L, Moeller B, Larry R, Misquez E (2010). Evidence-based practices in mentoring students with disabilities: four case studies. J Sci Educ Stud Disabil.

[18] Powers L, Sowers J, Stevens T (1995). An exploratory randomized study of the impact of mentoring on the self-efficacy and community-based knowledge of adolescents with severe physical challenges. J Rehabil.

[19] Shpigelman C, Weiss PL, Reiter S (2009). E-mentoring for all. Comput Hum Behav.

[20] Karcher MJ, Kuperminc GP, Portwood SG, Sipe CL, Taylor AS (2006). Mentoring programs: a framework to inform program development, research, and evaluation. J Community Psychol.

[21] O'Mally J, Antonelli K (2016). The effect of career mentoring on employment outcomes for college students who are legally blind. J Vis Impair Blind.

[22] Stinson JN, McGrath PJ, Hodnett ED, Feldman BM, Duffy CM, Huber AM, Tucker LB, Hetherington CR, Tse SML, Spiegel LR, Campillo S, Gill NK, White ME (2010). An internet-based self-management program with telephone support for adolescents with arthritis: a pilot randomized controlled trial. J Rheumatol.

[23] Fraas M, Bellerose A (2010). Mentoring programme for adolescent survivors of acquired brain injury. Brain Inj.

[24] Funnell MM (2010). Peer-based behavioural strategies to improve chronic disease self-management and clinical outcomes: evidence, logistics, evaluation considerations and needs for future research. Fam Pract.

[25] Sword C, Hill K (2003). Creating mentoring opportunities for youth with disabilities: issues and suggested strategies. Am Rehabil.

[26] Buckner A (1993). Mediating At-Risk Factors Among Seventh and Eighth Grade Students With Specific Learning Disabilities Using a Holistically Based Model [dissertation].

[27] Gregg N, Galyardt A, Wolfe G, Moon N, Todd R (2016). Virtual mentoring and persistence in STEM for students with disabilities. Career Dev Transit Except Individ.

[28] Cawthon SW, Johnson PM, Garberoglio CL, Schoffstall SJ (2016). Role models as facilitators of social capital for deaf individuals: a research synthesis. Am Ann Deaf.

[29] Barnard-Brak L, Schmidt M, Wei T, Hodges T, Robinson E (2013). Providing postsecondary transition services to youth with disabilities: results of a pilot program. J Postsecond Educ Disabil.

[30] Muscott H, O'Brien S (1999). Teaching character education to students with behavioral and learning disabilities through mentoring relationships. Educ Treat Child.

[31] Bierema L, Merriam S (2002). E-mentoring: using computer mediated communication to enhance the mentoring process. Innov High Educ.

[32] Leck J, Elliott C (2016). Shattering glass: e-mentoring and the advancement of diversity. Int J Organ Divers.

[33] Nguyen HQ, Carrieri-Kohlman V, Rankin SH, Slaughter R, Stulbarg MS (2004). Internet-based patient education and support interventions: a review of evaluation studies and directions for future research. Comput Biol Med.

[34] Eysenbach G, Powell J, Englesakis M, Rizo C, Stern A (2004). Health related virtual communities and electronic support groups: systematic review of the effects of online peer to peer interactions. BMJ.

[35] Cai RA, Beste D, Chaplin H, Varakliotis S, Suffield L, Josephs F, Sen D, Wedderburn LR, Ioannou Y, Hailes S, Eleftheriou D (2017). Developing and evaluating JIApp: acceptability and usability of a smartphone app system to improve self-management in young people with juvenile idiopathic arthritis. JMIR Mhealth Uhealth.

[36] Jibb LA, Stevens BJ, Nathan PC, Seto E, Cafazzo JA, Johnston DL, Hum V, Stinson JN (2018). Perceptions of adolescents with cancer related to a pain management app and its evaluation: qualitative study nested within a multicenter pilot feasibility study. JMIR Mhealth Uhealth.

[37] Bohleber L, Crameri A, Eich-Stierli B, Telesko R, von Wyl A (2016). Can we foster a culture of peer support and promote mental health in adolescence using a web-based app? A control group study. JMIR Ment Health.

[38] Breakey VR, Bouskill V, Nguyen C, Luca S, Stinson JN, Ahola Kohut S (2018). Online peer-to-peer mentoring support for youth with hemophilia: qualitative needs assessment. JMIR Pediatr Parent.

[39] Stinson J, McGrath P, Hodnett E, Feldman B, Duffy C, Huber A, Tucker L, Hetherington R, Tse S, Spiegel L, Campillo S, Gill N, White M (2010). Usability testing of an online self-management program for adolescents with juvenile idiopathic arthritis. J Med Internet Res.

[40] Lenhart A, Page D (2015). Teens, social media & technology overview 2015: smartphones facilitate shifts in communication landscape for teens.

[41] Sandhu S, Veinot P, Embuldeniya G, Brooks S, Sale J, Huang S, Zhao A, Richards D, Bell MJ (2013). Peer-to-peer mentoring for individuals with early inflammatory arthritis: feasibility pilot. BMJ Open.

[42] Hoey LM, Ieropoli SC, White VM, Jefford M (2008). Systematic review of peer-support programs for people with cancer. Patient Educ Couns.

[43] White M, Dorman SM (2001). Receiving social support online: implications for health education. Health Educ Res.

[44] Thompson L, Jeffries M, Topping K (2010). E‐mentoring for e‐learning development. Innov Educ Teach Int.

[45] Single PB, Single RM (2005). E‐mentoring for social equity: review of research to inform program development. Mentor Tutor.

[46] Cole DA, Nick EA, Zelkowitz RL, Roeder KM, Spinelli T (2017). Online social support for young people: does it recapitulate in-person social support; can it help?. Comput Human Behav.

[47] Shpigelman C, Gill CJ (2013). The characteristics of unsuccessful e-mentoring relationships for youth with disabilities. Qual Health Res.

[48] Kasprisin CA, Single PB, Single RM, Muller CB (2003). Building a better bridge: testing e-training to improve e-mentoring programmes in higher education. Mentor Tutor.

[49] Paloff R, Pratt K (1999). Building Learning Communities in Cyberspace: Effective Strategies for the Online Classroom.

[50] Gopalakrishnan S, Ganeshkumar P (2013). Systematic reviews and meta-analysis: understanding the best evidence in primary healthcare. J Family Med Prim Care.

[51] Petticrew M, Roberts H (2006). Systematic Reviews in the Social Sciences: A Practical Guide.

[52] Getchius TS, Moses LK, French J, Gronseth GS, England JD, Miyasaki J (2010). AAN guidelines: a benefit to the neurologist. Neurology.

[53] Kmet L, Lee R, Cook L (2004). Standard quality assessment criteria for evaluating primary research papers from a variety of fields.

[54] Liberati A, Altman DG, Tetzlaff J, Mulrow C, Gøtzsche PC, Ioannidis JPA, Clarke M, Devereaux PJ, Kleijnen J, Moher D (2009). The PRISMA statement for reporting systematic reviews and meta-analyses of studies that evaluate health care interventions: explanation and elaboration. J Clin Epidemiol.

[55] Ammerlaan J, van Os-Mendendorp H, de Boer-Nijhof N, Scholtus L, Kruize AA, van Pelt P, Prakken B, Bijlsma H (2017). Short term effectiveness and experiences of a peer guided web-based self-management intervention for young adults with juvenile idiopathic arthritis. Pediatr Rheumatol Online J.

[56] Gorter JW, Stewart D, Cohen E, Hlyva O, Morrison A, Galuppi B, Nguyen T, Amaria K, Punthakee Z, TRACE Study group (2015). Are two youth-focused interventions sufficient to empower youth with chronic health conditions in their transition to adult healthcare: a mixed-methods longitudinal prospective cohort study. BMJ Open.

[57] Ammerlaan J, van Os-Mendendrop H, Scholtus L, de Vos A, Zwier M, Bijlsma H, Kruize AA (2014). Feasibility of an online and a face-to-face version of a self-management program for young adults with a rheumatic disease: experiences of young adults and peer leaders. Pediatr Rheumatol Online J.

[58] Parkyn H, Coveney J (2013). An exploration of the value of social interaction in a boys' group for adolescents with muscular dystrophy. Child Care Health Dev.

[59] Stewart M, Barnfather A, Magill-Evans J, Ray L, Letourneau N (2011). Brief report: an online support intervention: perceptions of adolescents with physical disabilities. J Adolesc.

[60] Narad ME, Bedell G, King JA, Johnson J, Turkstra LS, Haarbauer-Krupa J, Wade SL (2018). Social Participation and Navigation (SPAN): description and usability of app-based coaching intervention for adolescents with TBI. Dev Neurorehabil.

[61] Cantrell K, Fischer A, Bouzaher A, Bers M (2010). The role of e-mentorship in a virtual world for youth transplant recipients. J Pediatr Oncol Nurs.

[62] Gregg N, Wolfe G, Jones S, Todd R, Moon N, Langston C (2016). STEM e-mentoring and community college students with disabilities. J Postsecond Educ Disabil.

[63] Todd R, Moon N, Langston C (2016). E-mentoring and its relevance for competency-based education for students with disabilities: research from the GSAA BreakThru model. Competency Based Educ.

[64] Burgstahler S, Cronheim D (2001). Supporting peer–peer and mentor–protégé relationships on the internet. J Res Technol Educ.

[65] Cohen K, Light J (2009). Use of electronic communication to develop mentor-protégé relationships between adolescent and adult AAC users: pilot study. Augment Altern Commun.

[66] Shpigelman C, Reiter S, Weiss PL (2009). A conceptual framework for electronic socio-emotional support for people with special needs. Int J Rehabil Res.

[67] Mastropieri MA, Scruggs TE, Klingerman K, Mohler L, Jeffs T, Boon R, Castellani J, Scruggs TE, Mastropieri MA (2001). University e-mail mentors for elementary students with disabilities: attitudinal and literacy effects. Advances in Learning and Behavioral Disabilities. Volume 15. Technological Applications.

[68] Bell EC (2010). Mentoring transition-age youth with blindness. J Spec Educ.

[69] Kim-Rupnow WS, Burgstahler S (2004). Perceptions of students with disabilities regarding the value of technology-based support activities on postsecondary education and employment. J Spec Educ Technol.

[70] Burgstahler S, Doyle A (2005). Gender differences in computer-mediated communication among adolescents with disabilities: a case study. Disabil Stud Q.

[71] Kramer JM, Ryan CT, Moore R, Schwartz A (2018). Feasibility of electronic peer mentoring for transition-age youth and young adults with intellectual and developmental disabilities: Project Teens making Environment and Activity Modifications. J Appl Res Intellect Disabil.

[72] Keane K, Russell M (2014). Using cloud collaboration for writing assignments by students with disabilities: a case study using action research. Open Praxis.

[73] Ahola Kohut S, Stinson JN, Ruskin D, Forgeron P, Harris L, van Wyk M, Luca S, Campbell F (2016). iPeer2Peer program: a pilot feasibility study in adolescents with chronic pain. Pain.

[74] Stinson J, Ahola Kohut S, Forgeron P, Amaria K, Bell M, Kaufman M, Luca N, Luca S, Harris L, Victor C, Spiegel L (2016). The iPeer2Peer Program: a pilot randomized controlled trial in adolescents with juvenile idiopathic arthritis. Pediatr Rheumatol Online J.

[75] Ahola Kohut S, Stinson J, Forgeron P, van Wyk M, Harris L, Luca S (2018). A qualitative content analysis of peer mentoring video calls in adolescents with chronic illness. J Health Psychol.

[76] Raghavendra P, Newman L, Grace E, Wood D (2013). 'I could never do that before': effectiveness of a tailored Internet support intervention to increase the social participation of youth with disabilities. Child Care Health Dev.

[77] Kim K, Choi H (2017). The influence of e-mentoring with college students with disabilities on career decision self-efficacy and college preparation for high school students with disabilities. Korean J Spec Educ.

[78] Smith CC, Cihak DF, Kim B, McMahon DD, Wright R (2017). Examining augmented reality to improve navigation skills in postsecondary students with intellectual disability. J Spec Educ Technol.

[79] Deci E, Ryan R (2008). Self-determination theory: a macro theory of human motivation, development and health. Can Psychol.

[80] Eisenman L (2007). Self-determination interventions: building a foundation for school completion. Remedial Spec Educ.

[81] Mumbardó-Adam C, Shogren KA, Guàrdia-Olmos J, Giné C (2016). Contextual predictors of self-determined actions in students with and without intellectual disability. Psychol Sch.

[82] Hohlfeld TN, Ritzhaupt AD, Dawson K, Wilson ML (2017). An examination of seven years of technology integration in Florida schools: through the lens of the Levels of Digital Divide in Schools. Comput Educ.

[83] Lindsay S, Evans C (2010). Disability and the digital divide: gaps and future directions. Internet Issues: Blogging, Digital Divide and Digital Libraries.

[84] Anderberg P (2007). Peer assistance for personal assistance: analysis of online discussions about personal assistance from a Swedish web forum for disabled people. Disabil Soc.

[85] Ragins BR, Cotton JL (1991). Easier said than done: gender differences in perceived barriers to gaining a mentor. Acad Manage J.

