# Supplementary material for: Electronic Mentoring Programs and Interventions for Children and Youth With Disabilities: Systematic Review
Source: JMIR Pediatr Parent. 2018 Oct 24;1(2):e11679. doi: 10.2196/11679 (PMC6716434; doi:10.2196/11679)
Supplement: Multimedia Appendix 1 [file pediatrics_v1i2e11679_app1.pdf]

Multimedia Appendix 1: Search strategy

Database: Ovid MEDLINE: Epub Ahead of Print, In-Process & Other Non-Indexed Citations, Ovid MEDLINE® Daily and Ovid MEDLINE® <1946-Present>, Embase Classic+Embase <1947 to 2018 May 18>, PsycINFO <1806 to May Week 2 2018>, Ovid Healthstar <1966 to April 2018>
Search Strategy:
----
1 exp disability/ or disability.mp. or exp physical disability/ (672955)
2 exp disabled person/ or exp disability/ or disab*.mp. (1013825)
3 cerebral palsy.mp. or exp cerebral palsy/ (80916)
4 spina bifida.mp. or exp spinal dysraphism/ (36554)
5 exp meningomyelocele/ or spinal dysraphism.mp. or exp neural tube defect/ or exp spinal dysraphism/ or exp spinal cord malformation/ (74187)
6 exp spine injury/ or exp spinal cord injury/ or spinal cord injur*.mp. or exp paraplegia/ (231197)
7 exp amputation/ or exp limb prosthesis/ or amput*.mp. (157682)
8 stroke.mp. or exp cerebrovascular accident/ (856723)
9 congenital anomal*.mp. (52317)
10 exp hydrocephalus/ or hydrocephalus.mp. (103423)
11 juvenile arthritis.mp. or exp juvenile rheumatoid arthritis/ (33634)
12 muscular dystrophy.mp. or exp muscular dystrophy/ (91021)
13 congenital malformation.mp. or exp congenital malformation/ (941226)
14 congenital malformation.mp. or exp congenital malformation/ (941226)
15 neuromuscular.mp. (198271)
16 exp autism/ or autism.mp. (166172)
17 brain injury.mp. or exp brain injury/ (334937)
18 blind.mp. or exp blindness/ (815605)
19 deaf.mp. or exp hearing impairment/ (209127)
20 deaf.mp. or exp hearing impairment/ (209127)
21 online.mp. (318687)
22 virtual.mp. (147608)
23 email.mp. or exp e-mail/ (45177)
24 computer.mp. or exp computer/ (2495092)
25 skype.mp. (1256)
26 internet.mp. or exp Internet/ (300243)
27 electronic mentor.mp. (0)
28 telementor.mp. (26)
29 peer.mp. (262171)
30 peer support.mp. or exp peer group/ (56825)
31 social support.mp. or exp social support/ (271399)
32 mentor.mp. or exp mentor/ (33208)
33 support group.mp. or exp support group/ (41327)
34 child/ (4129693)
35 youth.mp. or exp juvenile/ (3711754)
36 adolescent/ (4336897)
37 teen.mp. or exp adolescence/ (2934837)
38 limit 37 to (human and yr="1990 -Current") (2029452)
39 1 or 2 or 3 or 4 or 5 or 6 or 7 or 8 or 9 or 10 or 11 or 12 or 13 or 14 or 15 or 16 or 17 or 18 or 19 (4794016)
40 21 or 22 or 23 or 24 or 25 or 26 or 27 or 28 (3048495)
41 27 or 28 or 29 or 30 or 31 or 32 or 33 (580218)
42 34 or 35 or 36 or 37 (7619108)
43 39 and 40 and 41 and 42 (607)

44 remove duplicates from 43 (414)
