# Supplementary material for: Electronic Mentoring Programs and Interventions for Children and Youth With Disabilities: Systematic Review
Source: JMIR Pediatr Parent. 2018 Oct 24;1(2):e11679. doi: 10.2196/11679 (PMC6716434; doi:10.2196/11679)
Supplement: Multimedia Appendix 2 [file pediatrics_v1i2e11679_app2.pdf]

Multimedia Appendix 2. Overview of studies

Authors, Year (country)	Objectives	[a] Sample characteristics	[a] Disability type	Study Design (measures; theory)	Intervention (delivery and dosage)	Key findings (effect size)	Quality rating (AAN) and limitations
Ammerlaan et al. 2014 (Netherlands) [57]	To test the feasibility of an online and face-to-face self-management program	*Mentees:* 19 (aged 17-25; 84% female)<br>*Mentors:* 2 peer leaders (aged 20-30 with rheumatic disease)	Rheumatic disease	Mixed methods (perceived usefulness, ease of use, acceptance, adherence)<br>-Theory: Self-efficacy theory	-Password protected interactive website<br>-6 weeks ; weekly group chat (clarified goals, activities on weekly theme)<br>-Face-to-face version: 3 days<br>-Dosage: 12 hours	-Both the online and face-to-face programs were feasible and helped youth to deal with daily life<br>-Participants found the program easy to use	-Majority of sample was female<br>(IV)
Ammerlaan et al. 2017 (Netherlands) [55]	To investigate the effectiveness of a web-based self-management intervention guided by peer trainers	*Mentees:* 67 (aged 16-25; 35 intervention (83% female; mean 19.2); 32 control (94% female; mean age 19.1<br>*Mentors*: young adults with rheumatic disease	Juvenile arthritis	RCT and qualitative (Dutch arthritis self-efficacy scale, self-management, quality of life, medication use, learning and adherence<br>-Theory: self-efficacy theory	-Web-based self-management intervention with peer trainers (6 weeks)<br>-Group-based chat section (weekly), home exercises and discussion board<br>-Dosage: approx. 12 hours	-No significant differences on self-efficacy, quality of life, and self-management between experimental and controls<br>-Modeling and sharing were the most recognized themes in experimental group<br>-Goals included improving and maintaining balance, setting and recognizing boundaries, communicating and coping	-High proportion of females<br>-limited details of mentors<br>(I)
Barnfather et al. 2011 (Canada) [13]	To determine the extent to which adolescents used an online peer support intervention	*Mentees:* 27 aged 12-18 (mean 14.6) n=27 (15 boys; 12 girls)<br>*Mentors:* 5, same disability type; 22-39 years (3 women; 2 men)	Cerebral palsy, spina bifida	Survey and interviews (satisfaction, use and usefulness, social support;<br>-Theory: social support theory	-6-month online discussion forums<br>-Met once/week in group-based chat room for 1-1.5 hours per session for 25 sessions; email and message boards<br>-Ongoing monitoring and support<br>-Dosage 25-37.5 hours	-Two thirds of participants thought the intervention was fun<br>-Factors influencing the perceived utility of the intervention included typing speed, cognitive skills and need for support<br>-Females were significantly more	-Convenience sample<br>(IV)

						likely to contribute than males	
Bell 2010 (US) [68]	To explore the role of mentoring youth with blindness	*Mentees:* 49 (Aged 16-26; mean 21.1; 57% females) *Mentors:* adults with blindness	Legal blindness	Pre-post survey (career decision self-efficacy; Miller Hope Scale; social responsibility) -Theory: n/a	-Face-to-face activities; phone calls, e-mails, instant messaging (one-to-one) -Dosage: avg. 8 hours per month	-Youth who participated in the project had significant increases in career decision-making efficacy (large effect), positive personal hope for the future and positive attitudes about blindness (large effect)	-No control group -Do not know which aspects of the program contributed to the outcomes (IV)
Burgstahler & Cronheim 2001 (US) [64]	This study explores whether computer-mediated communication can be used to initiate and sustain peer-peer and mentor-protégé relationships in people with disabilities	*Mentees:* 49 high school students interested in STEM (20 females; 29 males) *Mentors:* 35 adults (10 females; 25 males; Do-IT scholars; in college)	Various (vision, hearing, learning)	Survey and focus groups (frequency of communication; what they liked and didn't like -Theory: social support theory	-Do-IT (Disabilities, opportunities, internet working and technology) -Using adaptive technology, internet and sharing resources -Meet face-to-face then online over 2 years -One-to-one and group-based -Dosage: unknown	-Peer-peer and mentor-protégé relationships perform similar functions; however, peer-peer relationships were more personal -Barriers to e-mentoring include: difficulty expressing feelings, lots of messages and technical difficulties	-Demographic details not provided (IV)
Burgstahler & Doyle 2005 (US) [70]	To explore gender differences in computer-mediated communication between youth with disabilities and adult mentors	*Mentees:* 40 (60% male) *Mentors:* 34 adults with experience in STEM	Various types	Content analysis of email messages -Theory: n/a	-Face-to-face and online (e-mail; part of larger DO-IT (Disabilities, opportunities, internetworking and technology) program -One-to-one and group-based -Dosage: approx. 2 years	-Males sought and provided information about technology and the internet -Females communicated more frequently overall and shared more personal information	-Convenience sample -Had a binary view of gender -unclear whether the mentors had a disability (IV)
Cantrell et al. 2010 (US) [61]	To explore the role of e-mentorship in virtual environments	*Mentees:*8 youth with pediatric transplants *Mentors*: 3 students (without disabilities) trained in child psychology	Pediatric transplant	Mixed methods (qualitative and online log data; -Theory: electronic socio-emotional support theory	-Zora virtual world offering psychoeducational support -Dosage: twice a week for 14 weeks (facilitated group-based activities)	-They discussed bullying, physical appearance, school and pain management -Mentors play an important role in engaging participants	-Limited description of the methods -Socio-demographics of participants not described (IV)

Cohen & Light 2009 (US) [65]	To develop, implement and conduct a preliminary evaluation of a small-scale mentorship program for youth who use AAC	*Mentees:* 4 (2 males, 2 females; mean age 18.2) *Mentors:* 4 adult AAC users (3 males, 1 female, mean age 36.2)	Cerebral palsy with communication disability (use AAC)	Content analysis of email messages, frequency and length of messages and goals attained; satisfaction survey -Theory: n/a	-Email communication (one-to-one; paired with mentor for 6 months); given instructions on how to structure the relationship -Researchers prompted to communicate regularly -Dosage: ranged from 10-21 weeks	-Mentoring by successful users of AAC may be effective in easing the transition of younger users of AAC -All pairs maintained consistent communication during the program -The frequency and length of communication may have been influenced by availability of mentor and quality of the match	-Small sample -Non-standardized measures (IV)
Gorter et al. 2015 (Canada) [56]	To assess use, utility and impact of transition interventions designed to support and empower self-management in youth with chronic conditions during transition to adult care	*Mentees:* 50 (42% male, mean age 17.9) *Mentors:* Occupational therapist with expertise in transition (identity, age and gender kept anonymous from mentees)	Various physical and developmental	4-year mixed-method prospective cohort (frequency of use, utility, impact of transition interventions, goals, interviews -Theory: n/a	-Youth KIT (organizational tool that includes goal setting activities) and online transition mentor (one-to-one and group-based chats and email) -Exposure: 12-47 months -Dosage: total -dosage unknown	85% of youth used the KIT at least once -40% of participants engaged in chats with the mentor -82% logged in at least once pre-transfer -Perceived utility of the intervention was modest	No control group -limited details on sampling strategy (II)
Gregg et al. 2016 (US) [62]	To understand the provision of e-mentoring to support the educational persistence of students with disabilities at college	*Mentees:* 4 (3 females, 1 male *Mentors:* 4 (2 males, 2 females); faculty, graduate students, business leaders with expertise in STEM (completed 2 online mentor training modules)	Various (learning, visual impairment, physical)	-Qualitative case study -Theory: n/a	-Mentors were matched based on interest and learning style -Virtual mentoring (digital voice or text-based communication platforms; virtual reality avatars) -Required to meet at least 10 times per semester, complete project modules; one-to-one -Dosage:	-Virtual environments and social media tool use varied depending on context, accessibility and practical considerations -STEM learning and emotional supports were enhanced when embedded in the practice of e-mentoring -5 persistent	-Small sample -Mentored pairs meet “at least 10 times per semester”, so no control over dosage per pair -Limited detail on interview data collection and analysis (IV)

					unknown	constructs (intention to persist, self-determination, self-advocacy, science affect, math affect) informed STEM outcomes	
Gregg et al. 2016 (US) [27]	To investigate the effectiveness of virtual mentoring for enhancing the persistence of secondary and post-secondary students with disabilities in STEM	*Mentees:* 189 (42% had mobility / orthopedic impairment)<br>*Mentors:* 33 (faculty, grad students, teachers, business leaders with expertise in STEM; 1 had a disability)	Various (learning, ADHD, autism)	Survey, online log data (aspiration, self-determination, self-advocacy, science self-efficacy, math self-efficacy; self-determination theory)	-4-year e-mentoring program (digital voice communication platform or text-based)<br>-Mentored pairs meet at least 10 times per semester, complete modules, participate in virtual group activities<br>-Virtual environments and social media platforms<br>-Dosage: unknown	-Improved self-determination and self-advocacy<br>-Virtual mentoring enhances the persistence of students with disabilities<br>-Math self-efficacy and math interest improvement were noted for majority of participants<br>-Greatest improvements for females and minority students	-Only 78% completed both the pre-post survey<br>-Mentored pairs meet "at least 10 times per semester", so no control over dosage per pair (IV)
Keane & Russell 2014 (US) [72]	To demonstrate how emerging technologies can bridge the psychological and communication space between students and instructors	*Mentees:* 1 (male)<br>*Mentors: 1* (adult writing coach)	Cerebral palsy	Case study<br>-Theory: n/a	-Cloud (*Google* app); *Google* voice (6 months) (one-to-one)<br>-Dosage: unknown	-Developed efficient coaching process adaptable for all student who need assistant at a distance<br>-Facilitated student's understanding of what changes were needed to school work	-Limited description of the role of the mentor<br>-Limited detail on interview data collection and analysis (IV)
Kim & Choi 2017 (Korea) [77]	To investigate the influence of e-mentoring program for students with disabilities on career	*Mentees:*16 high school students (aged 17-21, mean 17.8; 37.5% male)<br>*Mentors:* 16 university students with disabilities;	Various (physical disability, visual impairment, hearing impairment)	Pre-post survey and interviews (career decision self-efficacy scale)<br>-Theory: n/a	-8 sessions and online training (one-to-one email and online chat) for 1 hour per week<br>-Activity goals and feedback<br>-Dosage: 8 hours	-Significant improvements in career decision self-efficacy at the end of the program<br>-Participants had better understanding of colleges and majors they were	-Did not have control group (level IV)

	decision self-efficacy and college preparation	aged 20-27; mean age 22.8; 42.7% male) Received training				interested in, improved knowledge about admissions procedures	
Kim-Rupnow & Burgstahler 2004 (US) [69]	To explore perceptions of students with disabilities regarding the value of technology-based support activities on postseconda ry education and employment	*Mentees:* 75 (52% male; aged 18-26; majority under 23) *Mentors:* adults with experience in STEM	Various	Retrospective survey (motivations for post-secondary, employment, social skills, advocacy skills, self-esteem) -Theory: n/a	-Part of DO-IT mentor program (face-to-face and online (email) -One-to-one and group-based -Dosage: approx. 2 years	-participants reported short and long-term growth in their level of preparation for college and employment and increased self-advocacy and social skills -participants rated computer and internet use the most valuable activity for developing their social, academic or career skills	-Unclear whether mentors had a disability -No control group (IV)
Kohut et al. 2016 (Canada) [73]	To develop and test the feasibility, acceptability and impact of the iPeer2Peer program	*Mentees:* 28 (aged 12-18, mean 14.8); 93% female); 12 intervention, 16 control) *Mentors*: trained youth with chronic pain, aged 18-25 who have learned to successfully manage their pain	Chronic pain (neuropathi c, widespread , abdominal, secondary to chronic disease)	Pilot RCT, wait-list controls -Feasibility, adherence, satisfaction, recalled pain inventory, daily functioning (conceptual framework for peer support in health care)	-10 Skype video calls (one-to-one) (20-30 minutes) over 8 weeks -Modelling and reinforcement by peers -Dosage: 2.6 to 5 hours	-The iPeer2Peer program was feasible and acceptable provided that adolescents were given more time to complete all the calls -They liked the flexibility of skype calls -Youth in the intervention had significant improvement in self-management skills and coping efforts were more successful.	-Intensive intervention that requires significant time commitment -Small sample size -Mostly female sample (I)
Kohut et al. 2018 (Canada) [75]	To determine the topics of discussion during open-ended peer mentoring between youth with chronic	*Mentees:* 28 (93% female; mean age 14.8; 57% with juvenile arthritis) *Mentors*: 11 (90% female; 53% with juvenile arthritis; mean	Various (57% juvenile arthritis)	Qualitative content analysis (conceptual framework for peer support in health care) -Theory: n/a	-52 calls (7 mentor-mentee pairings) (part of pilot RCT) -Asked to meet 10 times via Skype (one-to-one) calls over the course of 2-3 months -Dosage: unknown	-Themes included: illness impact (relationships, school/work, self-identity, personal stories), self-management (adherence, transition to adult care, coping), non-illness related	-Majority female participants (IV)

	conditions	age 17.9)				issues (school goals, hobbies and social environments)<br>-Mentors provided informational, appraisal and emotional support to adolescents	
Kramer et al. 2018 (US) [71]	To explore the feasibility of e-mentoring for transition age youth with disabilities	*Mentees:* 42 (aged 14-22; 61% male)<br>*Mentors:* 9 (with a disability; mean age 22.6; 4 males; 5 females; mentors received training and ongoing supervision)	Intellectual and developmental	Pre-post survey; content analysis of calls<br>-Fidelity and engagement, feasibility and acceptability<br>-Theory: social learning theory	-One-to-one mentoring (instrumental e-mentoring calls, email, online chat)<br>-12 weeks, 8 calls which paralleled group session curriculum<br>-31 peer-mentoring dyads<br>-Dosage: unknown	Mentees attended 87% of calls and engaged during 94% of call objectives (engaged in practice problem-solving)<br>-Mentors achieved 87% of objectives<br>-Significant relationship between use of mentoring supports and fidelity<br>-Intervention is suitable and acceptable to mentees with disabilities	-Program was labour intensive and expensive (IV)
Mastropieri et al. 2001 (US) [67]	To explore the role of e-mentors for elementary students with disabilities	*Mentees:* 17 elementary students (82% male)<br>*Mentor:* university student without disability	Learning and cognitive disabilities	-analysis of e-mail messages<br>-Theory: n/a	-Email (one-to-one)<br>-Dosage: 3 months	-Significant improvement in written communication (# of sentences) from beginning to end of mentoring	-Little information given about the mentors<br>-Did not use standardized measures (IV)
Narad et al. 2018 (US) [60]	To describe the social participation and navigation (SPAN) program and report preliminary outcomes	*Mentees:* 4 (mean age 15; 3 females, 1 male)<br>*Mentors*: 4 (graduate students; weekly group supervision by clinical psychologist and OT)	Traumatic brain injury	Feasibility study pre-post (social and behavior functioning; feasibility and acceptability)<br>-Theory: n/a	-Mobile app, online didactic information (interactive website) and peer coaching;<br>-Met weekly with mentor (one-to-one) via video conference (Skype) developed goals and plans to achieve them<br>-4weeks<br>-Dosage: 1 hour a	-SPAN program was well received (feasible and good utility)<br>-Youth used the app to define and achieve goals<br>-Youth self-report measures medium to large effect)	-Small sample (IV)

					week for 4 weeks (total around 4 hours)		
Parkyn & Coveney 2013 (Australia) [58]	To explore the value of "MD Mafia" online group for youth with muscular dystrophy	*Mentees:* 12 (males aged 14-17; 12 parents) *Mentors:* adult (male occupational therapist)	Muscular dystrophy	Qualitative content analysis of discussion forum -Theory: symbolic interaction theory	-Password protected online interactive group activities and discussion forum matched to youth's age, interest and skills (1 hour x 4 times/year) -Dosage: 4 hours	-Intervention has a strong collective identity (reflecting ideals of masculinity) -Group provides opportunity for socialization in a safe environment	-Lack of details about the intervention -all male sample (IV)
Shpigelman et al. 2009 (Israel) [19]	To evaluate an e-mentoring intervention program providing social and emotional support for youth with disabilities	*Mentees:* 13 (aged 15-20 (mean 17.5; 5 males; 8 females) *Mentors:* 7 (2 males; 5 females with a disability; received 2-hour training; supervised by researchers)	Physical, emotional, behavioural, intellectual	Qualitative content analysis -Theory: electronic socio-emotional support (ESES)	-Youth were matched based on gender, shared interests and hobbies (one-to-one) -Intervention involved 4 stages (personal acquaintance, moderated communication, online activities, saying goodbye) taking 8 months -Dosage: send at least 2 messages per week via email for approx. 8 months.	-Findings support the usability and utility of an e-mentoring intervention and the ESES conceptual framework -Mentors sent mean of 38.8 messages compared to mean of 27.3 messages sent by mentees -Themes of the messages included perceptions, attitudes and experiences of mentors, the e-mentoring process, participants role in the process, feelings experienced and duration of the intervention	-Higher proportion of females -Small sample -No comparison group (IV)
Shpigelman & Gill 2013 (US) [47]	To explore the characteristics of unsuccessful e-mentoring relationships for youth with disabilities	*Mentees;* 6 aged 15-20 *Mentors:* 6 (aged 22-28; 67% female with a disability); Serve as role models; share life experiences and focus on positive	Developmental, physical, emotional, behavioral	Qualitative thematic analysis -Theory: n/a	-E-mentor intervention to provide social and emotional support for youth with disabilities through asynchronous (one-to-one) e-mail for 8 months (at least 2 messages per week and meet	-Unsuccessful mentoring was associated with a more formal style and distant tone; successful mentoring included informal and supportive style	-Training of mentors not mentioned -Small, sub-sample (IV)

		aspects of disability)			face-to face 3 times)<br>-Youth were matched to mentor based on gender, shared interests and hobbies<br>-Dosage: unknown		
Stewart et al. 2011 (Canada) [59]	To explore the perceptions of adolescents with physical disabilities in an online intervention	*Mentees:* 22 (aged 12-18)<br>*Mentors:* 5 (3 female, 2 male aged 22-39 with cerebral palsy or spina bifida)	Physical disability (cerebral palsy, spina bifida)	Survey and interviews (loneliness and social dissatisfaction scale, self-report coping scale, sense of community scale, self-perception profile and children's inventory of social support<br>-Theory: n/a	-Met weekly online in group-based chat rooms for 60-90 minutes for 25 group sessions over 6 months<br>-Topics included independent living, health concerns, bullying, making friends, career planning, travelling, sports and building relationships<br>-Dosage: 25 to 37.5 hours	-More contact with other teens; decreased loneliness;<br>-Significant increased acceptance and confidence; increase in sense of community<br>-On average youth attended 8.5 group sessions	-Pilot study; small sample<br>-No control group<br>-No use of verification procedures for qualitative data<br>(IV)
Stinson et al. 2016 (Canada) [74]	To examine the feasibility and acceptability of an online peer mentoring program (iPeer2Peer) for youth with juvenile arthritis	*Mentees:* 30 (aged 12-18, mean 14.3; 97% female)<br>Mentors: 6 (aged 16-25, mean 18.8, 83% female who successfully manage their juvenile arthritis, screened and trained (2.5 days)	Juvenile arthritis	Pilot RCT (wait-list controls) (feasibility, acceptability, self-management, self-efficacy, pain, social support and quality of life)<br>-Theory: n/a	-Connected 10 times (20-30 minutes/time) over 8 weeks using Skype (one-to-one) video calls<br>-Dosage: 2.5 to 5 hours	-Half of the participants completed the 10 calls within 8 weeks<br>-Average call length was twice the required amount<br>-Participants reported satisfaction with the program and would recommend to their peers<br>-Mean engagement level was 8.53/10<br>-Improvement in perceived ability to manage arthritis compared to controls<br>-There is a need for flexibility and individualization	-Majority female sample<br>(I)

						in the iPeer2Peer program	
Todd et al. 2016 (US) [63]	To explore the relevance of e-mentoring for competency-based education for students with disabilities	*Mentees:* 97 (secondary and post-secondary students) *Mentors:* “various backgrounds”	Various (physical, sensory, ASD, mobility, other)	Pre-post survey (communication methods, interactions, self-advocacy, self-determination, intention to persist, science and math anxiety) -Theory: theory of change	-Georgia STEM accessibility alliance uses virtual worlds, online and smartphone technologies; individual and group activities -4 years (surveyed twice each year) -Dosage: unknown	-Participants reported higher frequency of using communication platforms that they are already acquainted with including e-mail, telephone, SMS text message -Improvements in internal characteristics related to persistence in STEM education -Self-advocacy had the highest gain	-No control group -Limited socio-demographic details of participants -Limited details on who the mentors are and what training they received (IV)

[a] Note: only the findings related to the objectives of this review were reported.
