# Supplementary material for: Electronic Mentoring Programs and Interventions for Children and Youth With Disabilities: Systematic Review
Source: JMIR Pediatr Parent. 2018 Oct 24;1(2):e11679. doi: 10.2196/11679 (PMC6716434; doi:10.2196/11679)
Supplement: Multimedia Appendix 3 [file pediatrics_v1i2e11679_app3.pdf]

Multimedia Appendix 3. Overall scores using the Standard Quality Assessment Criteria [53] for quantitative studies

Study	1	2	3	4	5	6	7	8	9	10	11	12	13	14	Total
Ammerlaan et al. 2014 [57]	2	1	1	2	NA	NA	NA	1	0	0	0	0	0	1	**8/22 0.36**
Ammerlaan et al. 2017 [55]	2	2	2	2	2	0	0	2	2	2	2	1	2	2	**23/28 0.82**
Bell 2010 [68]	2	1	1	2	NA	NA	NA	2	2	1	2	1	2	2	**18/22 0.81**
Cantrell et al. 2010 [61]	1	1	1	0	NA	NA	NA	1	1	NA	NA	0	0	1	**6/18 0.33**
Cohen & Light 2009 [65]	2	2	1	2	NA	NA	NA	2	1	NA	NA	1	0	1	**12/18 0.66**
Gorter et al. 2015 [56]	1	1	1	2	NA	NA	NA	2	2	2	2	NA	2	2	**17/20 0.85**
Gregg et al. 2016 [27]	2	1	1	1	NA	NA	NA	2	1	2	0	NA	2	2	**14/20 0.70**
Kim & Choi 2017 [77]	2	1	2	2	NA	NA	NA	2	1	2	1	1	2	2	**18/22 0.81**
Kim-Rupnow & Burgstahler 2004 [69]	2	1	1	1	NA	NA	NA	1	2	1	1	1	2	2	**15/22 0.68**
Kohut et al. 2016 [73]	2	2	2	2	2	NA	NA	2	2	2	2	1	2	2	**23/24 0.96**
Mastropieri et al. 2001 [67]	2	1	1	1	NA	NA	NA	1	1	1	0	0	1	2	**11/22 0.50**
Narad 2018 [60]	2	2	1	1	NA	NA	NA	2	1	2	1	1	2	1	**16/22 0.73**
Stewart 2011 [59]	2	1	1	1	NA	NA	NA	2	1	1	2	1	1	2	**15/22 0.68**
Stinson et al. 2016 [74]	2	1	2	1	2	2	NA	2	2	1	2	2	2	2	**23/26 0.88**

[a] Scoring: 2= yes, 1= partial, 0=no, n/a= not applicable

[b] Note: checklist for assessing quality of qualitative studies see Kmet et al. [53] for full description of items.
