# Supplementary material for: Electronic Mentoring Programs and Interventions for Children and Youth With Disabilities: Systematic Review
Source: JMIR Pediatr Parent. 2018 Oct 24;1(2):e11679. doi: 10.2196/11679 (PMC6716434; doi:10.2196/11679)
Supplement: Multimedia Appendix 4 [file pediatrics_v1i2e11679_app4.pdf]

Multimedia Appendix 4. Overall scores using the Standard Quality Assessment Criteria [53] for qualitative studies

Study	1	2	3	4	5	6	7	8	9	10	Total
Ammerlaan et al. 2014 [57]	2	1	1	2	1	2	1	0	2	2	**14/20 0.70**
Ammerlaan et al. 2017 [55]	2	2	1	2	1	2	2	2	2	1	**17/20 0.85**
Barnfather et al. 2011 [13]	2	2	1	2	1	2	2	2	2	0	**16/20 0.80**
Burghstahler and Cronheim 2001 [64]	2	2	2	2	1	2	2	2	1	1	**17/20 0.85**
Burgstahler and Doyle 2005 [70]	2	1	1	1	1	1	1	0	2	1	**11/20 0.55**
Cantrell et al. 2010 [61]	2	1	2	2	1	1	1	0	1	0	**11/20 0.55**
Cohen & Light 2009 [65]	2	2	1	2	2	2	1	2	1	0	**15/20 0.75**
Gregg et al. 2016 [62]	2	2	2	2	2	1	1	2	2	0	**16/20 0.80**
Keane and Russell 2014 [72]	2	1	2	1	0	0	0	0	1	0	**7/20 0.35**
Kohut et al. 2018 [75]	2	2	2	2	1	1	1	0	2	1	**14/20 0.70**
Kramer et al. 2018 [71]	2	2	2	2	1	1	1	2	2	0	**15/20 0.75**
Parkyn & Coveney 2013 [58]	2	2	1	2	2	2	2	2	2	0	**17/20 0.85**
Shpigelman et al. 2009 [19]	2	2	1	2	1	2	2	2	2	0	**16/20 0.80**
Shpigelman and Gill 2013 [47]	2	1	1	0	1	1	2	2	1	0	**11/20 0.55**
Stewart 2011 [59]	2	2	2	0	1	1	1	0	1	0	**10/20 0.50**
Todd et al. 2016 [63]	2	1	1	2	1	1	1	2	2	0	**13/20 0.65**

[a] Scoring: 2= yes, 1= partial, 0=no, n/a= not applicable

[b] Note: checklist for assessing quality of qualitative studies see Kmet et al. [53] for full description of items.
