# Supplementary material for: Electronic Mentoring Programs and Interventions for Children and Youth With Disabilities: Systematic Review
Source: JMIR Pediatr Parent. 2018 Oct 24;1(2):e11679. doi: 10.2196/11679 (PMC6716434; doi:10.2196/11679)
Supplement: Multimedia Appendix 5 [file pediatrics_v1i2e11679_app5.pdf]

Multimedia Appendix 5. Overview of components of electronic mentor interventions by level of evidence

Level of evidence	Intervention components	Outcomes
Level I	Interactive website (group-based) [55]<br><br>Skype calls[73, 74]	No significant differences on self-efficacy, quality of life, and self-management<br><br>Feasible and acceptable<br>Significant improvement in self-management skills and coping
Level II	Online mentor (group, 1 to 1)[56]	Utility of the intervention was modest
Level III		
Level IV	Email (one-to-one)[19, 47, 64, 65, 67-71,77]<br><br>Interactive website (group-based)[13, 57-60]<br><br>Virtual world[27, 61-63]<br><br>App[60, 63, 72]<br><br>Skype calls[60, 75]<br><br>Phone calls[68, 71]	Improved transition to using assistive devices<br>Increased career decision-making efficacy<br>Improved attitudes about disability<br>Improved written communication<br>Improved preparation for college and employment<br><br>Intervention is feasible, fun<br>Improved coping with daily life<br>Decreased loneliness, increased acceptance, confidence and sense of community<br>Provided strong collective identity and space for socialization<br>Participants more inclined to speak with mentors than peers<br><br>Improved engagement with psychoeducational goals<br>Enhanced science, technology and math learning and emotional supports<br>Improved persistence in science, technology and math and self-advocacy<br><br>Feasible and acceptable<br>Helped youth to define and achieve goals<br>Facilitate student understanding of school work<br><br>Intervention is feasible and acceptable<br>Increased goal attainment<br><br>Improved career decision-making, hope for the future and attitudes towards disability

		Intervention is feasible and acceptable

[a] Note: Level 1 (rigorous randomized control trials (RCTs)) and 2 (matched prospective cohort studies or RCTs in a representative population lacking 1 criteria in level 1); level 3 (all other RCTs); level 4 (studies not meeting 1-3)
